# Mental health service use in a national sample of college students with co-occurring depression or anxiety and substance use

**DOI:** 10.1016/j.dadr.2022.100025

**Published:** 2022-01-11

**Authors:** Samantha G. Auty, Sarah K. Lipson, Michael D. Stein, Sharon Reif

**Affiliations:** a Department of Health Law, Policy and Management, Boston University School of Public Health, 715 Albany Street, Boston, MA 02118, USA; b Heller School for Social Policy and Management, Institute for Behavioral Health, Brandeis University, 415 South Street, Waltham, MA 02453, USA

**Keywords:** Mental health, Mental health services, Substance-related disorders, Students

## Abstract

**Introduction:**

Campus health systems can provide timely and accessible resources for students with co-occurring substance use and mental illness, but little is known about the degree to which students use these systems. This study examined mental health service utilization among students with symptoms of anxiety or depression, stratified by substance use.

**Methods:**

This cross-sectional study used data came from the 2017–2020 Healthy Minds Study. Mental health service use was examined among students with clinically significant anxiety or depression (*N* = 65,969), stratified by substance use type (no use, alcohol or tobacco use, marijuana use, other drug use). We performed a series of weighted logistic regressions to assess the adjusted association of substance use type with past year use of campus, off-campus outpatient, emergency department, and hospital mental health services.

**Results:**

Among students, 39.3% reported exclusive use of alcohol or tobacco, 22.9% reported use of marijuana, and 5.9% reported use of other drugs. Use of alcohol or tobacco was not associated with mental health service utilization, while students who use marijuana faced increased odds of campus (OR 1.10, 95% CI 1.01, 1.20) and off-campus outpatient mental health service utilization (OR 1.27, 95% CI 1.17, 1.37). Other drug use was associated with increased odds of off-campus outpatient (OR 1.28, 95% CI 1.14, 1.48), emergency department (OR 2.13, 95% CI 1.50, 3.03) and hospital service utilization (OR 1.52, 95% CI 1.13, 2.04).

**Conclusions:**

Universities should consider screening for substance use and common mental illnesses to support the health of high-risk students.

## Highlights

• 84% of students with anxiety or depression perceived need for mental health services but only 37.8% accessed any mental health services.
• Students who use certain drugs (i.e. stimulants, opioids, etc.) used emergency and hospital services more than three times as often as other students.
• Universities should scale-up services for students with co-occurring mental illness and substance use.

## 1 Introduction

The traditional university years (ages ∼18–25) represent a period of initiation and intensification of substance use for many of the 22 million students enrolled in U.S. postsecondary education (Auerbach et al., 2016; Kessler et al., 2005). Alcohol, cannabis, and tobacco are the most commonly used substances among university students (Walters et al., 2018), but illicit use of other drugs including stimulants, hallucinogens, and opioid pain medications is becoming increasingly common (Substance Abuse and Mental Health Services Administration, 2019). From 2015 to 2019, use of illicit substances among young adults increased from 37.4% to 39.1% (Substance Abuse and Mental Health Services Administration, 2019). Use of substances among university students is associated with a myriad of negative outcomes including truancy and lower grade point averages (Arria et al., 2013b), sexual assault (Yeater et al., 2020), and self-injurious behavior (Serras et al., 2010) among others.

The increasing prevalence of substance use among young adults is particularly concerning given a concomitant increase in the prevalence of anxiety and depression in this population (Lipson et al., 2019). One-third of today's university students meet clinical criteria for anxiety or depression, and in the last three years, the prevalence of co-occurring mental illness and illicit substance use has increased by 22% (Lipson et al., 2019; Substance Abuse and Mental Health Services Administration, 2019). Over the same time period, use of any mental health services increased only 7% (Eisenberg and Lipson, 2019). A wide body of evidence suggests that substance use and mental illness often co-occur and have a synergistic relationship, wherein one can intensify the other (Arria et al., 2013a; Walters et al., 2018). Some evidence suggests that mental illness increases the risk of transitioning to misuse of substances and subsequent development of substance use disorders (SUD) (Conway et al., 2016). Furthermore, young adults with co-occurring mental illness and substance use are at increased risk for poor physical and mental health outcomes including chronic disorders, functional impairment, and suicide (King et al., 2020; Lipson et al., 2019; Lynch et al., 2020; Miller et al., 2016). Despite these risks, less than half of students with clinically-significant symptoms of common mental health disorders (i.e. depression and anxiety) receive any treatment (Bourdon et al., 2020; Cranford et al., 2009; Garcia-Williams et al., 2014; Hyun et al., 2006). One study found that among students with co-occurring substance use and mental illness, 67% reported a need for mental health care but only 38% accessed professional services (Cranford et al., 2009). Thus, the gap between need and access to mental health services among students with co-occurring mental illness and substance use is a significant problem.

A large body of research has examined the role of alcohol and marijuana use among students with co-occurring mental illness with respect to mental health service use, but relatively little research has examined the role of other drugs. Numerous studies have found that students with mental illness and comorbid hazardous alcohol or marijuana use more often seek out mental health services than those without (Capron et al., 2018; Cranford et al., 2009; Mathews et al., 2019; Pedrelli et al., 2016; Ramo et al., 2013). However, very little research has examined the impact of illicit use of prescription medications (i.e. stimulants, benzodiazepines) or other drugs (i.e. cocaine, hallucinogens) on mental health service use among students. Research has found that university students with SUDs are less likely to access mental health services than non-students peers (Blanco et al., 2008), but the majority of students who use substances do not have SUDs and likely differ in important ways that may impact mental health service use. Despite rising rates of substance use among students, no research has examined the role of substance use type in mental health service use among this group nationally. Moreover, no research has examined the types of mental health services used by university students with co-occurring mental illness and substance use in the last decade.

University health systems have the potential to serve as an entry point for students struggling with co-occurring mental illness and substance use. Examining patterns of mental health service utilization among students with symptoms of common mental health disorders and substance use is critical to identify opportunities for early intervention and identify treatment pathways for this understudied, high-risk, and growing population. As such, population-level research is needed to fill gaps in knowledge and to inform the optimization of campus health service delivery systems. This study examines the role of substance use type in mental health service use in a national sample of university students with clinically significant symptoms of generalized anxiety or major depression, the most common mental health disorders affecting this population. Specifically, we examined the association of substance use type with campus mental health service use and use of mental health services in the community.

## 2 Methods

### 2.1 Data

This cross-sectional study used three years of data collected during the school year from the Healthy Minds Study (HMS): Fall 2017-Spring 2018, Fall 2018-Spring 2019, and Fall 2019-Winter 2020 (Eisenberg and Lipson, 2019). The HMS is an annual, national, cross-sectional survey conducted online that examines mental health, substance use, and service utilization among undergraduate and graduate students in the U.S. The data are publicly available and de-identified. The sample of institutions included in the HMS is geographically diverse, and all institutions are eligible to participate regardless of size. At institutions with more than 4000 students, a random sample of 4000 students are invited to participate in the survey. At institutions with less than 4000 students, all students are invited to participate (Eisenberg et al., 2019). To account for non-response bias, the HMS research group constructs sample probability weights using administrative data on student characteristics (i.e. sex, race, academic level, and grade point average) from participating institutions (Lipson et al., 2019). These weights are constructed using logistic regressions to estimate the probability of response for each variable (Eisenberg et al., 2019; Lipson et al., 2019). Additional details on the sampling methodology used by the HMS and the construction of sample probability weights are available in the HMS annual report (Eisenberg et al., 2019).

Due to the COVID-19 pandemic and subsequent disruption in the delivery of mental health services, HMS data from the 2021 academic year was not included. The COVID-19 pandemic resulted in the closure of universities across the U.S., making campus mental health systems difficult or impossible for students to access (Son et al., 2020). The disruption in access to campus mental health services stemming from the COVID-19 pandemic would decrease use of these services irrespective of student characteristics, thus we focus our analysis on data collected prior to the onset of the pandemic from Fall 2017 to Winter 2020.

### 2.2 Study sample

The sample was restricted to those respondents who met criteria for clinically significant anxiety or depression and were enrolled in either undergraduate or graduate degree programs (*N* = 65,969, 36.0% of all respondents during the study period). We elected to limit the sample to those with clinically significant symptoms of depression or anxiety to examine the association between substance use and mental health service utilization among those with symptoms of common mental health disorders, a high-risk and growing population of students. The Patient Health Questionnaire-9 (PHQ-9) (Kroenke et al., 2001) was used to assess symptoms of major depression, and the Generalized Anxiety Disorder-7 (GAD-7) (Spitzer et al., 2006) was used to assess symptoms of anxiety. Both the PHQ-9 and the GAD-7 measure symptoms during the past two weeks. Clinically significant depression was defined as receiving a score of ≥10 on the PHQ-9, and clinically significant anxiety was defined as receiving a score of ≥10 on the GAD-7. Per prior research, a score of ≥10 on either the PHQ-9 and GAD-7 are indicative of moderate symptoms (Eisenberg et al., 2018, 2019; Lipson et al., 2019).

### 2.3 Measures

#### 2.3.1 Primary independent variable

The primary independent variable was substance use type. We opted to categorize by type of substances used for several reasons. First, students infrequently reported use of certain substances (i.e. opioids, methamphetamines), and small cell sizes prevented these substances from being evaluated individually in adjusted models. Second, the HMS survey did not collect data on substance use frequency or evaluate the presence of SUDs during the study period. For these reasons, we opted to categorize substance use by types of substances used.

Categorization of substance use was based on responses to multiple survey questions inquiring about past two week use of alcohol, and past month use of tobacco, marijuana, stimulants, cocaine, heroin, ecstasy, opioids, hallucinogens, and methamphetamines (see Appendix A for survey questions). These survey questions specifically inquire about non-prescribed use of substances (i.e. non-prescribed used of benzodiazepines or opioids). Use of other substances could be noted in a free-text response. We generated binary indicator variables for use illicit use of other drugs (i.e. benzodiazepines, hallucinogens, etc.) and electronic cigarette use or other tobacco use through this item, which were frequently reported in this manner.

Specifically, we measured substance use as follows: Non-substance users reported no use of any substances; alcohol and tobacco users reported only use of either or both substances; marijuana users reported use of marijuana exclusively or in addition to tobacco and alcohol use; other drug users reported illicit or non-prescribed use of stimulants, cocaine, opioids, ecstasy, hallucinogens, or methamphetamines exclusively or in addition to tobacco, alcohol, and/or marijuana use. Other drug and tobacco indicator variables generated from free-text responses contributed to counts of each substance, respectively. We opted to evaluate marijuana separately due to the changing regulatory landscape across the U.S. and the higher frequency of use among young adults (34.8%) in comparison to the other illicit substances, which remain illegal across states in the U.S. and are used less frequently (Lancione et al., 2020; Substance Abuse and Mental Health Services Administration, 2019).

Substance use was coded as a factor variable, where non-substance users were coded as “0,” alcohol or tobacco users were coded as “1,” marijuana users were coded as “2,” and other drug users were coded as “3.” This method assigned respondents to the highest level of substance use they reported. For example, a student who reported use of alcohol and cocaine would be considered an other drug user, and a student who reported use of alcohol and marijuana would be considered a marijuana user.

#### 2.3.2 Primary outcome variables

The primary outcome variables were any past-year use of campus mental health services, off-campus outpatient services (i.e. therapist, psychiatrist, other mental health provider), emergency department, and hospital mental health services (partial hospitalization and inpatient hospitalization). Evaluation of past-year mental health service use was based on response to one survey question: *From which of the following places did you receive counseling or therapy?* Respondents could endorse use of multiple service types in response to this question (i.e. use of emergency department *and* outpatient services). All outcomes were measured as binary variables, where “0″ indicated no past-year use of a given service and “1″ indicated past-year use of a given service (see Appendix A for survey questions).

#### 2.3.3 Secondary outcome variables

We also evaluated barriers to accessing mental health services in the past year. Barriers included financial limitations, not having enough time, unsure of where to seek care, difficulty finding an available appointment, and preferring to deal with issues on own or with friends (see Appendix A for survey questions). Barriers to mental health services were also evaluated as binary variables, taking a value of “0″ if a barrier was not endorsed and “1″ if it was.

#### 2.3.4 Covariates

We examined respondent characteristics that were included in all waves of the HMS survey during the study period. Demographic characteristics included age, gender (male, female, transgender, genderqueer/other), race (white, Asian, Black, Latinx, Middle Eastern, other), sexual orientation (heterosexual, gay/lesbian, bisexual, queer/other), degree program (undergraduate vs. graduate degree program), health insurance type (uninsured, student, parent, individual, other), grade point average (above 3.0, below 3.0, or unknown) and living place (i.e. on-campus housing, social housing, off-campus housing, or other housing). In addition to these variables, we also included PHQ-9 and GAD-7 scores as covariates to assess differences in the severity of depression and anxiety symptoms. We also included a binary measure of perceived need for help with mental health problems in the past year.

### 2.4 Statistical analysis

We first assessed differences in respondent characteristics across substance use groups using weighted *t*-tests for continuous variables and chi-square tests for categorical variables. All bivariate estimates are presented as weighted percentages or means with standard deviations. We performed a series of weighted logistic regressions to assess the association between past-year mental health service utilization by type and substance use group. Logistic regressions were adjusted for age, gender, race, sexual orientation, degree program, living place, insurance type, PHQ-9 score, GAD-7 score, and perceived need for mental health services. Due to small cell sizes, we reduced the levels of race and health insurance in adjusted analyses (see Appendix B). Adjusted models also included university fixed effects to account for time-invariant differences between universities, and year fixed effects to control for secular trends. To account for correlation among errors, we used robust standard errors in all models. All model estimates are presented as odds ratios (OR) with 95% confidence intervals (CI). Additionally, the marginal effect (ME) of substance use group was calculated for each model, which expresses the change in the probability that the outcome occurs (i.e. past year mental health service use type) when substance use type changes while holding other covariates constant (Norton et al., 2019; Williams, 2012). To assess model fit, we also performed Hosmer–Lemeshow goodness-of-fit tests for all models. All analyses were performed in Stata/MP 16.1.

## 3 Results

### 3.1 Sample characteristics

Sample characteristics stratified by substance use group are presented in Table 1. Among respondents with clinically significant symptoms of either depression or anxiety, 39.3% were tobacco and or alcohol users, 22.9% were marijuana users, and 5.9% were other drug users. Other drug users were more frequently male (44.7%) than marijuana users (36.7%), alcohol or tobacco users (33.2%), and non-users (31.2%). Other drug users were also more often white (70.4%) compared to marijuana users (65.0%), alcohol or tobacco users (66.5%), and non-users (54.7%). Residence also differed between substance use groups; other drug users more frequently lived in social housing (6.9%) than marijuana (3.5%), alcohol or tobacco (2.5%), and non-users (1.6%). Students who reported use of other drugs had higher PHQ-9 scores (*M* = 15.5, SD = 5.1) than students using marijuana (*M* = 14.5, SD = 5.1), tobacco or alcohol (*M* = 12.0, SD 4.8), or no substances (*M* = 13.8, SD = 5.2). In this sample of students with clinically significant anxiety or depression, students reporting no substance use less frequently perceived a need for mental health services (80.9%) than alcohol or tobacco users (83.8%), marijuana users (89.0%), and other drug users (89.9%).

**Table 1:** Weighted sample characteristics of HMS respondents from 2017—2020 with depression or anxiety, stratified by substance use.

Characteristics	Non-Users (22,156)	Alcohol or Tobacco Users (n = 27,334)	Marijuana Users (14,686)	Other Drug Users (n = 3,494)
	M(SD) or %			
Age	21.7 (5.5)	22.7 (5.4)	21.4 (3.9)	21.7 (3.9)
Gender				
Male	31.2%	33.2%	36.7%	44.7%
Female	63.9%	63.9%	58.4%	51.2%
Transgender	1.4%	0.7%	1.2%	0.7%
Genderqueer/other	3.5%	2.2%	3.6%	3.4%
Race				
White	54.7%	66.5%	65.0%	70.4%
Asian	16.3%	10.7%	6.9%	6.2%
Latinx	10.7%	10.0%	10.4%	9.9%
Black	10.0%	6.6%	9.7%	5.3%
Middle eastern	3.6%	2.0%	2.3%	2.5%
Other	4.8%	4.2%	5.7%	5.7%
Sexual Orientation				
Heterosexual	69.8%	72.9%	60.8%	64.5%
Gay or lesbian	4.6%	4.8%	5.8%	4.5%
Bisexual	11.1%	11.5%	17.6%	16.4%
Queer or other	14.4%	10.9%	15.8%	14.6%
Degree Program				
Bachelors	86.1%	79.5%	88.3%	89.3%
Graduate	13.9%	20.5%	11.7%	10.7%
Grade Point Average				
> 3.0	76.3%	80.4%	76.0%	76.7%
≤ 3.0	10.5%	10.3%	13.2%	14.3%
Unknown	13.2%	9.2%	10.8%	9.0%
Living Place				
On-campus housing	45.1%	36.2%	44.0%	39.0%
Social housing	1.6%	2.5%	3.5%	6.9%
Off-campus housing	27.6%	44.0%	39.4%	42.5%
Other	25.7%	17.3%	13.2%	11.6%
Insurance Type				
Uninsured	5.0%	4.1%	4.6%	4.9%
Student	10.2%	10.9%	9.5%	8.5%
Parent	5.8%	5.9%	64.2%	65.7%
Individual	7.5%	10.3%	5.8%	6.0%
Other	19.0%	15.3%	15.9%	14.9%
PHQ-9 Score	13.8 (5.2)	13.5 (5.2)	14.5 (5.1)	15.5 (5.1)
GAD-7 Score	11.9 (4.9)	12.0 (4.8)	12.2 (4.8)	12.3 (4.7)
Perceived Need for Services	80.9%	83.8%	89.0%	89.9%

*Proportions may not add to 100 due to rounding.

Substance use frequency between groups is presented in Table 2. Rates of alcohol use were very high in all groups, but highest among students who reported use of only alcohol or tobacco (94.5%). Other drug users reported tobacco use most frequently (65.3%), but a substantial proportion of marijuana users (44.3%) and tobacco or alcohol users (20.9%) also reported use. Other drug users most commonly used stimulants (58.6%), cocaine (36.1%), opioids (19.1%), and ecstasy (12.2%). The least commonly used drugs among other drug users were hallucinogens (6.4%) and methamphetamines (2.6%).

**Table 2:** Use of substances by substance use group among students with depression or anxiety.

Characteristics	Alcohol or Tobacco Users (n = 27,334)	Marijuana Users (14,686)	Other Drug Users (n = 3,494)
Alcohol	94.5%	81.6%	88.9%
Tobacco	20.9%	44.3%	65.3%
Marijuana	-	100%	78.2%
Stimulants	-	-	58.6%
Cocaine	-	-	36.1%
Opioids	-	-	19.1%
Ecstasy	-	-	12.2%
Hallucinogens	-	-	6.4%
Methamphetamines	-	-	2.6%

**Notes:** Alcohol and tobacco users reported only use of either or both substances. Marijuana users reported use of marijuana exclusively or in addition to tobacco and alcohol use. Other drug users reported illicit or non-prescribed use of stimulants, cocaine, opioids, ecstasy, hallucinogens, or methamphetamines exclusively or in addition to tobacco, alcohol, and/or marijuana use.

### 3.2 Mental health service utilization

Across the entire sample, 37.8% (*N* = 26,259) used any mental health services in the past year. Past year utilization of any mental health services was highest among other drug users (49.1%) and marijuana users (49.1%), while a smaller proportion of alcohol or tobacco (39.6%) and non-users (38.2%) reported use of any services (*p* < 0.001) (Table 3). While only 21.1% (*N* = 14,154) of the sample utilized campus mental health services, utilization was significantly higher among marijuana (25.8%) and other drug users (24.2%) in comparison to alcohol or tobacco users (21.0%) and non-users (21.0%) (*p* < 0.001). Use of off-campus outpatient mental health services was 23.8% (*N* = 17,022) across the entire sample. Other drug and marijuana users utilized off-campus outpatient mental health services at higher levels, 29.3% and 29.1%, respectively, than alcohol or tobacco (22.8%) and non-users (21.6%) (*p* < 0.001). Across the entire sample, 1.4% (*N* = 843) reported use of the emergency department for mental health needs. Other drug users reported past year utilization of the emergency department for mental health needs nearly twice as often (3.2%) as marijuana users (1.7%), and nearly three times as often alcohol or tobacco users (1.1%) and non-users (1.2%) (*p* < 0.001). Use of hospital mental health services was 1.8% (*N* = 1189) across the entire sample; 3.5% of students reporting use of other drugs utilized hospital mental health services in comparison to only 2.3% of marijuana users, 1.4% of alcohol or tobacco users, and 1.7% of non-users (*p* < 0.001).

**Table 3:** Past year utilization of mental health services among students with depression or anxiety, stratified by substance use.

Characteristics	Non-Users (22,156)	Alcohol or Tobacco Users (n = 27,334)	Marijuana Users (14,686)	Other Drug Users (n = 3,494)	*P* value
Any	38.2%	39.6%	49.1%	49.1%	<0.001
Campus services	20.5%	21.0%	25.8%	24.2%	<0.001
Off-campus outpatient services	21.6%	22.8%	29.1%	29.3%	<0.001
Emergency department	1.2%	1.1%	1.7%	3.2%	<0.001
Psychiatric hospital services	1.7%	1.4%	2.3%	3.5%	<0.001

**Notes:** The HMS inquires about emergency department use specifically for psychiatric or mental health needs.

### 3.3 Odds of mental health service use

Adjusted results examining mental health service utilization outcomes are presented in Table 4. In comparison to students who did not report use of any substances, alcohol or tobacco use was not associated with utilization of campus mental health services, off-campus outpatient mental health services, the emergency department, or hospital mental health services after adjustment.

**Table 4:** Adjusted odds of past-year mental health services utilization among students with depression or anxiety.

**Variable**	Campus Services OR (95% CI)	Off-Campus Services OR (95% CI)	ED Services OR (95% CI)	Hospital Services OR (95% CI)
Substance use (vs. no use)				
Alcohol or tobacco use	0.99 (0.92, 1.07)	0.99 (0.92, 1.06)	0.92 (0.70, 1.20)	0.78 (0.52, 1.02)
Marijuana use	1.10 (1.01, 1.20)	1.27 (1.17, 1.37)	1.10 (0.85, 1.44)	1.07 (0.85, 1.34)
Other drug use	1.06 (0.94, 1.24)	1.28 (1.14, 1.48)	2.13 (1.50, 3.03)	1.52 (1.13, 2.04)
PHQ-9 score	1.02 (1.01, 1.03)	1.02 (1.01, 1.02)	1.09 (1.06, 1.10)	1.08 (1.06, 1.10)
GAD-7 score	1.01 (1.01, 1.02)	1.02 (1.01, 1.02)	1.03 (1.01, 1.05)	1.01 (0.98, 1.02)
Perceived need	8.50 (7.00, 10.32)	5.55 (4.88, 6.31)	15.0 (7.39, 30.44)	5.83 (3.18, 10.70)
Graduate degree program (vs. Bachelor)	0.97 (0.88, 1.07)	0.99 (0.91, 1.08)	0.51 (0.36, 0.74)	0.68 (0.52, 0.90)
Residence (vs. on-campus)				
Social housing	1.11 (0.90, 1.36)	0.82 (0.67, 0.98)	0.71 (0.36, 1.41)	0.74 (0.45, 1.29)
Off-campus	0.72 (0.66, 0.77)	0.95 (0.88, 1.02)	1.02 (0.80, 1.32)	1.01 (0.81, 1.25)
Other	0.43 (0.39, 0.48)	0.97 (0.89, 1.06)	1.27 (0.95, 1.71)	1.26 (0.96, 1.65)
Health insurance (vs. uninsured)				
Student	1.27 (1.06, 1.52)	1.80 (1.49, 2.19)	2.54 (1.34, 4.83)	2.02 (1.14, 1.77)
Parent	0.98 (0.84, 1.15)	2.36 (1.51, 2.49)	2.68 (1.50, 4.79)	2.49 (1.52, 4.10)
Other	0.72 (0.61, 0.85)	1.93 (1.63, 2.30)	2.66 (1.49, 4.78)	2.14 (1.29, 3.54)
Gender (vs. male)				
Female	1.17 (1.09, 1.27)	1.46 (1.36, 1.56)	1.21 (0.94, 1.57)	1.42 (1.25, 2.54)
Transgender	2.04 (1.57, 2.67)	1.94 (1.51, 2.49)	2.48 (1.41, 4.37)	2.12 (1.24, 3.63)
Genderqueer and other	1.45 (1.22, 1.72)	1.76 (1.49, 2.08)	0.86 (0.54, 1.37)	1.68 (1.08, 2.62)
Sexual orientation (vs. heterosexual)				
Gay or lesbian	1.52 (1.33, 1.75)	1.51 (1.33, 1.71)	1.47 (0.99, 2.19)	1.79 (1.25, 2.54)
Bisexual	1.39 (1.29, 1.51)	1.44 (1.33, 1.55)	1.91 (1.49, 2.45)	1.81 (1.48, 2.21)
Other	1.70 (1.58, 1.86)	1.55 (1.43, 1.69)	1.93 (1.45, 2.56)	1.68 (1.34, 2.12)
Race (vs. white)				
Asian	1.02 (0.92, 1.13)	0.47 (0.43, 0.52)	1.14 (0.80, 1.63)	0.65 (0.47, 0.89)
Latinx	0.89 (0.81, 0.98)	0.72 (0.66, 0.79)	0.91 (0.64, 1.28)	0.77 (0.58, 1.03)
Black	1.07 (0.94, 1.21)	0.51 (0.46, 0.58)	0.84 (0.54, 1.31)	0.53 (0.38, 0.75)
Other	0.93 (0.82, 1.06)	0.74 (0.66, 0.82)	1.03 (0.73, 1.47)	1.15 (0.83, 1.58)
Age	0.98 (0.98, 0.99)	1.04 (1.03, 1.05)	1.03 (1.01, 1.05)	1.01 (0.99, 1.03)

Notes: ED – emergency department. All models include university and year fixed effects.

After adjustment, marijuana use was associated with increased odds of both campus (OR 1.10, CI 1.01, 1.20) and off-campus outpatient mental health service utilization (OR 1.27, CI 1.17, 1.37). Holding all other covariates constant at their observed values, this translates to a 1.6 percentage point increase in the probability of campus mental health service utilization (CI 0.3, 2.9) and a 4.1 percentage-point increase in the probability of off-campus mental health service utilization (CI 2.7, 5.4). Marijuana use was not associated with either utilization of the emergency department for mental health needs or hospital services after adjustment.

Other drug use was associated with increased odds of off-campus mental health service utilization (OR 1.28, CI 1.14, 1.48), translating to a 4.4 percentage point increase in the probability of utilization (CI 2.1, 6.6). Other drug was also associated with increased odds of both emergency department (OR 2.13, CI 1.50, 3.03) and hospital mental health service utilization (OR 1.52, CI 1.13, 2.04), respectively translating to a 1.6 (CI 0.6, 2.2) and 1.1 (CI 0.4. 1.8) percentage-point increase in the probability of using emergency department and hospital services. However, other drug use was not associated with campus mental health service utilization after adjustment. Hosmer–Lemeshow goodness-of-fit tests indicated that the fit of all models was satisfactory (i.e. *p* > 0.30). Several demographic and psychological characteristics were also associated with mental health service use, and the full results are presented in Table 4.

### 3.4 Barriers to mental health services

**Fig. 1:**
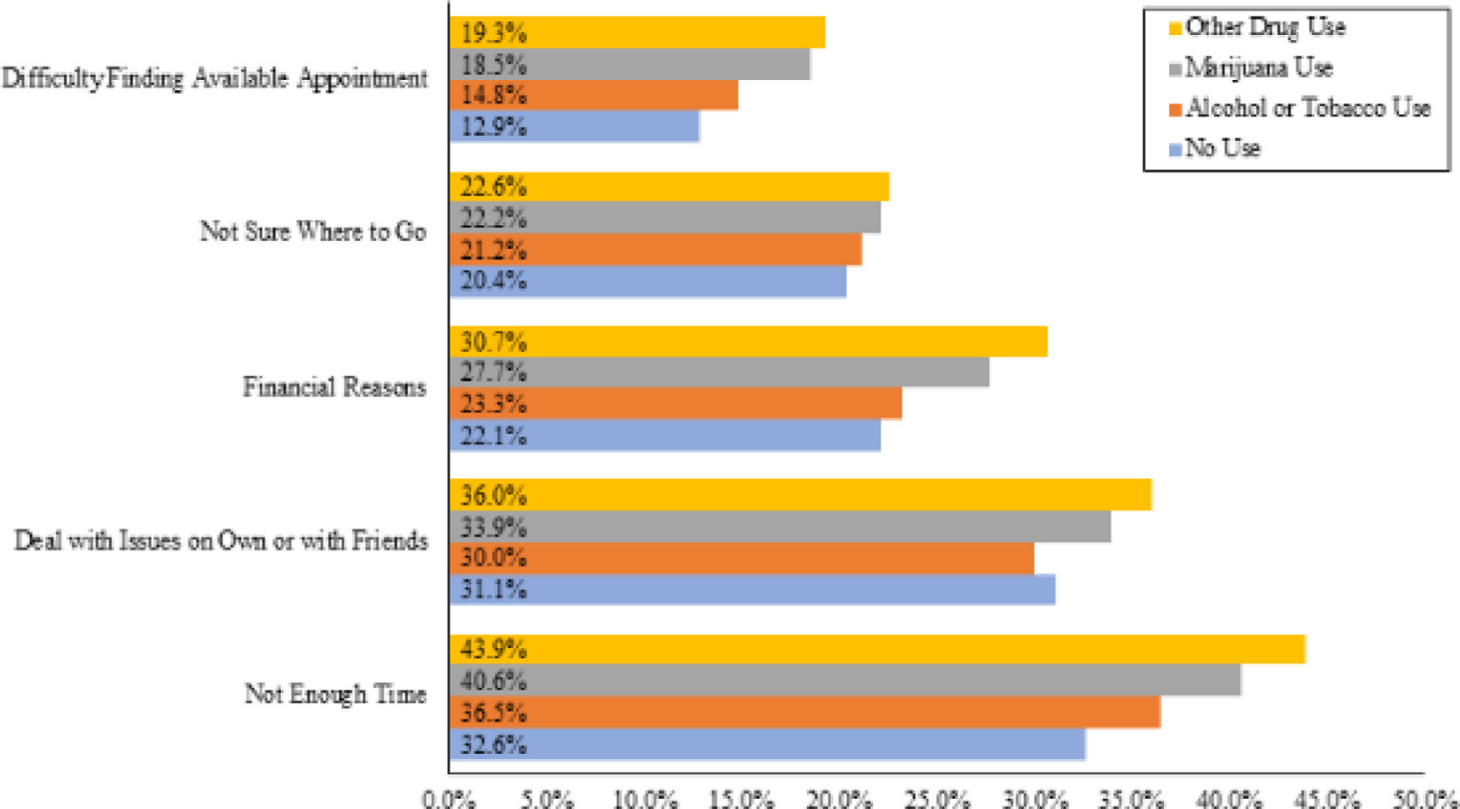
Percent of students reporting barriers to mental health services stratified by substance use group.

Other drug users more often reported barriers to mental health services than all other substance use groups (Fig. 1). Students who reported use of other drugs more often endorsed financial reasons (30.7%) as a barrier to services than marijuana users (27.7%), alcohol or tobacco users (23.2%), and non-users (22.1%) (*p* < 0.001). Other drug users also more often reported that they preferred deal with mental health issues on their own or to seek help from family or friends (36.0%) in comparison to marijuana users (33.9%), alcohol or tobacco users (30.0%), and non-users (31.1%) (*p* < 0.001). Having not enough time for mental health services was the most commonly reported barrier by other drug users (43.9%), and these students more often reported this as a barrier to care then students who used marijuana (40.6%), alcohol or tobacco (36.5%), or non-substance users (32.6%) (*p* < 0.001).

## 4 Discussion

This is the first large-scale study to have examined mental health service utilization among university students with co-occurring substance use and symptoms of common mental health disorders. In this study of students with clinically significant symptoms of depression or anxiety, 84% of students reported need for mental health services but only 38.2% of students accessed any services. We found that students who use other drugs (i.e. illicit or non-prescribed use of stimulants, cocaine, opioids, ecstasy, hallucinogens, or methamphetamines exclusively or in addition to tobacco, alcohol, and/or marijuana use) and have symptoms of depression or anxiety, utilize emergency department and hospital services more often than students who exclusively use alcohol, tobacco, or marijuana with these symptoms. These findings demonstrate a critical need to identify and provide appropriate services to students with co-occurring mental illness and other drug use, which carries risk for lifetime development of SUDs (Lynch et al., 2020; Salom et al., 2016). While many campuses have taken steps to better meet the needs of students with mental health illness, additional solutions may be needed to improve the identification and care of vulnerable students.

Students with common mental health disorders more frequently use substances than those without, which suggests that some may be self-medicating symptoms with substances (Walters et al., 2018; Zullig and Divin, 2012). This is particularly concerning given that increased use of substances may worsen symptoms of mental illness, and intensify this pattern (Andrews and Westling, 2016; Colder et al., 2013; Zullig and Divin, 2012). This study found that other drug use was associated with an increased the likelihood of emergency department and hospital service use for mental health needs, potentially indicating that using certain substances heightens the mental health needs of these students. While higher rates of emergency and hospital service use may be reflective of more severe mental health problems (Fleury et al., 2019), it may also reflect an inadequate dose of outpatient mental health care (Frosch et al., 2011). Students across all groups reported similar use of outpatient services, but students who used other drugs had higher PHQ-9 and greater perceived need for mental health services than all other groups. Students using other drugs may not receive an appropriate mix of services to support their mental health needs, potentially leading to increased use of emergency and hospital services.

While the campus mental health system may be ideally positioned to provide outpatient mental health care, previous research has demonstrated that university students frequently seek support from nonprofessional sources (Eisenberg et al., 2011, 2012). In this study, students who reported use of other drugs reported more barriers to care but also used most types of mental health services more frequently than other substance groups, potentially indicating unmet need for certain services. Moreover, other drug users more often reported that they preferred to seek help from family or friends than other groups (Fig. 1). Stigma related to substance use is pervasive, and can be a significant barrier to accessing professional treatment services (Crapanzano et al., 2019; Hammarlund et al., 2018). Thus, students who use certain substances may perceive less stigma when discussing problems with peers. This preference may also reflect fears of university-sponsored consequences related to substance use. Use of illicit substances is generally prohibited on university campuses, and use of substances can jeopardize athletic participation or scholarships (Buckner et al., 2018). Thus, students who use certain substance may prefer to seek support from peers or from providers not affiliated with their university. Universities should consider peer gatekeeper training, which trains non-healthcare professionals (i.e. residence life advisors) to link those in distress with appropriate resources. Trained peers may be a beneficial addition to campus resources for those who prefer to seek care from non-professional sources, and may ultimately facilitate access to professional care.

The COVID-19 has spurred increasing rates of both mental illness and substance use among young adults (Firkey et al., 2021; Lechner et al., 2020; Son et al., 2020; Wang et al., 2020). Many students have returned to university campuses, and universities should consider ways to support students who may be struggling with intensified substance use or mental illness. While campus mental health systems may be unable to meet the needs of students with more severe mental illness, they are ideally positioned to link these students to appropriate resources. Care coordination is partially reliant on the appropriate identification of those in need of services. The use of screeners, like the Screening, Brief Intervention, and Referral to Treatment, may facilitate identification and referral to treatment for those students in need of care (Farmer et al., 2019). Universities should also consider gatekeeper training and student education on the intersection of substance use and mental health. Given the increasing prevalence of mental illness and substance use, there is a growing and urgent need for integrated behavioral health services within campus health systems to meet the needs of these high-risk students (Hesse, 2009; Lydecker et al., 2010).

## 5 Limitations

While this study is strengthened by random selection of students and the multisite methodology of HMS, there are several elements that limit the generalizability of findings. First, while the survey employs weights to account for non-response bias, low response rates are a notable limitation (2017-2018: 23%, 2018-2019: 16%, 2019-2020: 14.5%). While non-response weights can adjust for known characteristics, students who elected not to complete HMS may differ from those that did in important ways that influence mental health service use (i.e. more severe substance use or mental health problems). Second, schools elect to participate in HMS, and while schools are diverse in composition and geographic location, they are not randomly selected. Additionally, students who elect to complete the survey may different than those who do not. However, we apply non-response weights to mitigate this selection bias. Third, past-month substance use may not accurately capture patterns or the intensity of substance use. Additionally, we are unable to determine if substance use precedes mental health service utilization. While these limitations warrant consideration, the HMS is a valuable data source to examine national trends in substance use and mental health service utilization that would otherwise be difficult to assess.

## 6 Conclusions

Decreasing the gap between need , access, and utilization for students with co-occurring illness and substance use is critically important to avoid unnecessary morbidity. Future research should examine the role of stigma among students, which may impact access to mental health care. Institutions should consider methods to identify and support these students, which may include increased use of routine screening and investment in care coordination with off-campus resources.

## Author disclosures

The authors have nothing to disclose.

## Funding

This work was supported by NIDA P30DA035772 (Auty, Reif), NIDA 5T32DA041898-03 (Auty), NIMH 1K01MH121515 (Lipson), William T Grant Foundation Scholars Program (Lipson).

## CRediT authorship contribution statement

**Samantha G. Auty:** Conceptualization, Formal analysis, Data curation, Writing – original draft, Writing – review & editing, Visualization, Funding acquisition. **Sarah K. Lipson:** Conceptualization, Formal analysis, Writing – original draft, Writing – review & editing. **Michael D. Stein:** Conceptualization, Writing – review & editing, Supervision. **Sharon Reif:** Conceptualization, Writing – review & editing, Supervision.

## Declaration of Competing Interest

The authors have no conflicts of interest to disclose.

## Appendix Supplementary materials

Image, application 1
