# Supplementary material for: Mental health service use in a national sample of college students with co-occurring depression or anxiety and substance use
Source: Drug Alcohol Depend Rep. 2022 Jan 11;2:100025. doi: 10.1016/j.dadr.2022.100025 (PMC9948943; doi:10.1016/j.dadr.2022.100025)
Supplement: Supplementary file 1 [file mmc1.docx]

**Appendix A. Select survey items and response options**

| **Measure** | **Survey Item** | **Survey Response** |
| --- | --- | --- |
| Lifetime mental health service utilization | Have you ever received counseling or therapy for mental health concerns? | 1=No, never  2=Yes, prior to starting college  3=Yes, since starting college  4=Yes, both of the above (prior to college and since starting) |
| Past year campus mental health service utilization | From which of the following places did you receive counseling or therapy? (Select all that apply) | 1 = Name of institutions student counseling services  1 = Name of institutions campus health services  1 = Name of institutions other campus counseling or health services |
| Past year private mental health service utilization | From which of the following places did you receive counseling or therapy? (Select all that apply) | 1=Provider in the local community (not on campus) |
| Past year psychiatric emergency room utilization | From which of the following places did you receive counseling or therapy? (Select all that apply) | 1 = Psychiatric emergency services/emergency room |
| Past year psychiatric inpatient hospital utilization | From which of the following places did you receive counseling or therapy? (Select all that apply) | 1 = Inpatient psychiatric hospital |
| Past year psychiatric partial hospitalization utilization | From which of the following places did you receive counseling or therapy? (Select all that apply) | 1 = Partial hospitalization program |
| Barriers to services | In the past 12 months, which of the following factors have caused you to receive fewer services (counseling, therapy, or medications) for your mental or emotional health than you would have otherwise received? (Select all that apply) | 1 = Financial reasons (too expensive, not covered by insurance)  1 = Not enough time  1 = Not sure where to go  1 = Difficulty finding an available appointment time  1 = Prefer to deal with issues on my own or with support from family/friends |
| Past month substance use | Over the past 30 days, have you used any of the following drugs? (Select all that apply) | 1 = Marijuana  1 = Cocaine  1 = Heroin  1 = Opioid pain relievers  1 = Methamphetamines  1 = Other stimulants  1 = Ecstasy  1 = Other drugs |
| Past month cigarette use | Over the past 30 days, about many cigarettes did you smoke per day? | 1=0 cigarettes  2=Less than 1 cigarette  3=1 to 5 cigarettes  4=About one-half pack  5=1 or more packs |
| Alcohol use | Over the past 14 days, did you drink any alcohol? | 1 = Yes  2 = No |

**Appendix B. Covariate levels in bivariate and multivariate analyses**

| **Measure** | **Bivariate Levels** | **Multivariate Levels** |
| --- | --- | --- |
| Sexual orientation | 1. Heterosexual 2. Gay or lesbian 3. Bisexual 4. Queer 5. Other | 1. Heterosexual 2. Gay or lesbian 3. Bisexual 4. Other |
| Race | 1. White 2. Asian 3. Hispanic 4. Black 5. Middle eastern 6. Other | 1. White 2. Asian 3. Hispanic 4. Black 5. Other |
| Health insurance type | 1. Parent 2. Student 3. Employer sponsored 4. Government sponsored 5. Other 6. Uninsured | 1. Uninsured 2. Parent 3. Student 4. Other |
| Living place | 1. On-campus housing 2. Greek or social housing 3. Off-campus housing 4. Family 5. Other | 1. On-campus 2. Greek or other social housing 3. Off-campus 4. Other |
